# 

**Published:** 1916-04-29

**Authors:** 


### Personalia.

Mr. Robert J. Gourlay has been appointed treasurer of Greenock Infirmary in succession to the late Mr. John Rodger.

Miss Hilda K. Brade has been appointed assistant director of the Clinical Laboratory of the Manchester Royal Infirmary.

Dr. C. J. Bennett, of Buxton, the medical officer of health for the Chapel-en-le-Frith Rural District, has retired after forty-four years' service.

Dr. T. Lauder Thomson, county medical officer, has been requested by the General Purposes Committee of the Dumbarton County Council not to apply for a commission in the R.A.M.C.

Dr. Henry G. Sneeth, chairman of the Stockport Corporation Health Committee, serving with the R.A.M.C., has been promoted temporary lieutenant-colonel.

Dr. Anthony Vost, of Stirling, has been appointed medical officer of health for that burgh. Dr. Thomas Adam, the present medical officer for Stirlingshire, has been offered the post of tuberculosis officer.

Sir Edward Fithian has resigned the hon. secretaryship of the London Temperance Hospital owing to failing health. At the unanimous wish of his colleagues on the board of management the office has been accepted by Mr. George Blaiklock, J.P., Recorder of Grantham.

Rates of Subscription: Twelve months, 6/6; Six months, 4/-; Three months, 2/-, post free to any address in the United Kingdom. Abroad, Twelve months, 8/8; Six months, 5/-; Three months, 2/6, post free.—Address The Subscription Dept., "The Hospital," The Hospital Building, 28/29 Southampton Street, Strand, London, W.C.

